# Periampullary cancer and neurological interactions: current understanding and future research directions

**DOI:** 10.3389/fonc.2024.1370111

**Published:** 2024-03-19

**Authors:** Yuchen Wang, Zi’ang Liu, Yanzhang Tian, Haoliang Zhao, Xifeng Fu

**Affiliations:** ^1^ Third Hospital of Shanxi Medical University, Shanxi Bethune Hospital, Shanxi Academy of Medical Sciences, Tongji Shanxi Hospital, Taiyuan, China; ^2^ General Surgery Department , Shanxi Bethune Hospital/General Surgery Department, Third Hospital of Shanxi Medical University, Taiyuan, China; ^3^ Tongji Hospital, Tongji Medical College, Huazhong University of Science and Technology, Wuhan, China

**Keywords:** periampullary cancer, perineural invasion, pancreatic cancer, ampulla of Vater, nerve

## Abstract

Periampullary cancer is a malignant tumor occurring around the ampullary region of the liver and pancreas, encompassing a variety of tissue types and sharing numerous biological characteristics, including interactions with the nervous system. The nervous system plays a crucial role in regulating organ development, maintaining physiological equilibrium, and ensuring life process plasticity, a role that is equally pivotal in oncology. Investigations into nerve-tumor interactions have unveiled their key part in controlling cancer progression, inhibiting anti-tumor immune responses, facilitating invasion and metastasis, and triggering neuropathic pain. Despite many mechanisms by which nerve fibers contribute to cancer advancement still being incompletely understood, the growing emphasis on the significance of nerves within the tumor microenvironment in recent years has set the stage for the development of groundbreaking therapies. This includes combining current neuroactive medications with established therapeutic protocols. This review centers on the mechanisms of Periampullary cancer’s interactions with nerves, the influence of various types of nerve innervation on cancer evolution, and outlines the horizons for ongoing and forthcoming research.

## Introduction

Periampullary cancer refers to a malignancy that occurs around the ampulla of Vater, encompassing several types such as pancreatic head cancer, ampullary cancer, distal common bile duct adenocarcinoma, and duodenal adenocarcinoma (1). The ampulla of Vater is a wider part formed after the convergence of the bile and pancreatic ducts, which ultimately leads into the small intestine. Due to its unique location, periampullary cancer can cause obstruction of the common bile duct and the main pancreatic duct even at an early stage of tumor growth, leading to jaundice in patients.

In the clinical setting, PACs are relatively scarce, constituting a mere 5% of all gastrointestinal malignant tumors (2). Within the spectrum of gastrointestinal malignancies, PACs are ranked between the eighth and ninth in terms of incidence, and are also the fourth to fifth leading causes of death associated with gastrointestinal malignancies. The majority of PACs are adenocarcinomas, such as pancreatic cancer and bile duct adenocarcinoma, among others. Additionally, other rare tumor types, including islet cell or neuroendocrine tumors, papillary cystic tumors, lymphomas, acinar cell tumors, and pancreatic metastases originating from other primary sites, can also be found in the periampullary region. These uncommon tumors usually demonstrate more favorable prognoses (3). Periampullary tumors, a group characterized by diverse biological behaviors and prognoses, exhibit varying molecular features and survival rates. Despite substantial advancements in disease staging and adjuvant therapies over the past three decades, there has been no significant improvement in the long-term survival rates for any type of periampullary cancer.

Pancreaticoduodenectomy (PD) has been widely accepted as the standard treatment for periampullary cancers. Clinical and pathological factors that influence long-term survival of patients after PD include patient age, CA19-9 level, carcinoembryonic antigen (CEA) level, tumor size, location, histological differentiation, lymph node metastasis, vascular invasion, and perineural invasion (PNI) (4). Among them, elevated levels of CA19-9, larger tumor size, poorer histological differentiation, lymph node metastasis, and PNI are considered independent prognostic factors for adverse outcomes in patients with pancreatic adenocarcinoma (PAC) after surgery (5, 6). Particularly noteworthy is PNI, which has received widespread attention in recent years as an independent risk factor for PAC prognosis.

Solid tumors display heterogeneous perineural involvement, which can arise from cancer cells enveloping normal nerve structures or inducing neoneurogenesis through the secretion of neurotrophic factors. Pathologically, PNI is characterized by the infiltration and dissemination of cancer cells along the nerve sheath or surrounding tissues, with tumor cells encircling at least 120 degrees of the nerve (5). PNI is considered one of the most prominent forms of interaction between tumor cells and nerve cells within the tumor microenvironment. Clinically, PNI can lead to surgical challenges, impair sensitivity to radiation therapy and chemotherapy, and serve as a significant factor contributing to the progression and adverse prognosis of various malignant tumors (6).

Currently, research on PNI primarily focuses on molecular mechanisms and pathophysiology, revealing that PNI can exist independently of lymph node metastasis or vascular invasion. This may be attributed to direct interactions or signaling between nerve cells and tumor cells within the surrounding. The tumor microenvironment is currently a prominent area of research, with the recognition of the tumor as a holistic entity being widely accepted. It is understood that the tumor microenvironment, along with all cells influencing tumor cell growth, collectively contribute to the characteristics of tumor cells. This review aims to explore the intricate interactions between neuronal cells, tumor cells, and other stromal cells within the tumor microenvironment, elucidating the diverse pathways involved in tumor development and the consequential impact on tumor phenotype.

## Neuroanatomical features surrounding the pancreatic head

**Figure 1:**
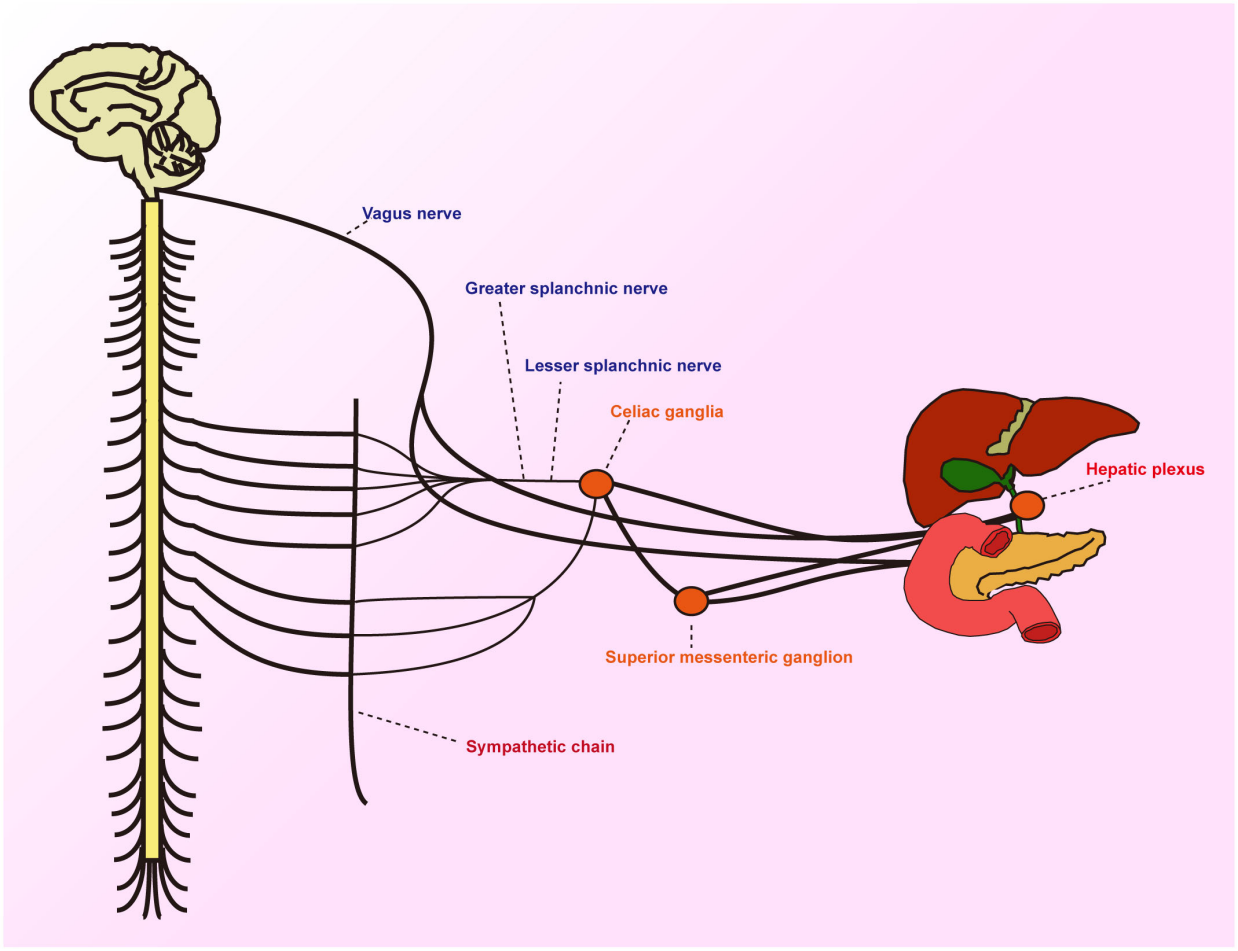
Schematic diagram showing the periphery of the hepatobiliary and pancreatic jugular innervated by sensory, sympathetic and parasympathetic fibres.

The head of the pancreas and its surrounding areas boast a rich neural network, encompassing the pancreatic head plexus, abdominal plexus, liver plexus, aortic plexus, splenic plexus, and the plexus surrounding the superior mesenteric artery. These neural plexuses, composed of sympathetic and parasympathetic nerves, play a pivotal role in pancreatic secretion and pain transmission (7). Changes in the pancreatic head plexus are closely linked to early postoperative liver metastasis and mortality. Pancreatic cancer frequently metastasizes along these neural pathways, with pancreatic head cancer most likely to invade the plexus around the superior mesenteric artery, whereas cancers of the body and tail tend to invade the splenic plexus (8). The sympathetic nerves of the pancreas primarily originate from the anterior roots of the thoracic section of the greater splanchnic nerve and accompany the branches of the portal vein and hepatic artery. The parasympathetic nerves mainly stem from the hepatic branch of the vagus nerve, which, along with the common bile duct, enters the wall of the duodenum and then branches into the pancreas. The neural input to the pancreas is primarily provided by the spinal cord, vagus nerve, enteric nervous system, and either dorsal root ganglia or nodose ganglia (9). The normal functioning of the pancreas relies on the integrated action of these nerves (**Figure 1**).

The intricate neural network in the periampullary region provides tumors with close proximity to nerves, thereby increasing the risk of neural invasion. Observations indicate that even though both belong to the category of cholangiocarcinomas, there exists a significant difference in the rate of neural invasion between intrahepatic and extrahepatic anatomical locations (10). Nerves influence the pathophysiological behaviors of tumor cells through various mechanisms, and in turn, they are affected by neurotrophic factors produced by the tumor and its microenvironment. This bidirectional interaction further intensifies both neural dysfunction and tumor progression. Given the complex interplay between tumors and nerves, we classified the effects of nerves on Periampullary cancer as follows: promoting tumor cell proliferation, resistance to death, inhibiting anti-tumor immunity, aiding in invasion and metastasis, and showcasing nerve damage within the tumor-inflammatory setting. These effects are intricately correlated to the hallmarks observed in tumor cells, such as enhanced proliferation, increased resistance to death, evasion from immune oversight, heightened invasiveness, potential for metastasis, and Tumor-Promoting Inflammation (11). It’s crucial to emphasize that these traits encapsulate the collective behavior of the tumor tissue as a whole. Given the pivotal role of neural tissues in the tumor milieu, their influence cannot be underestimated.

## Neural promotion of tumor cell proliferation

**Figure 2:**
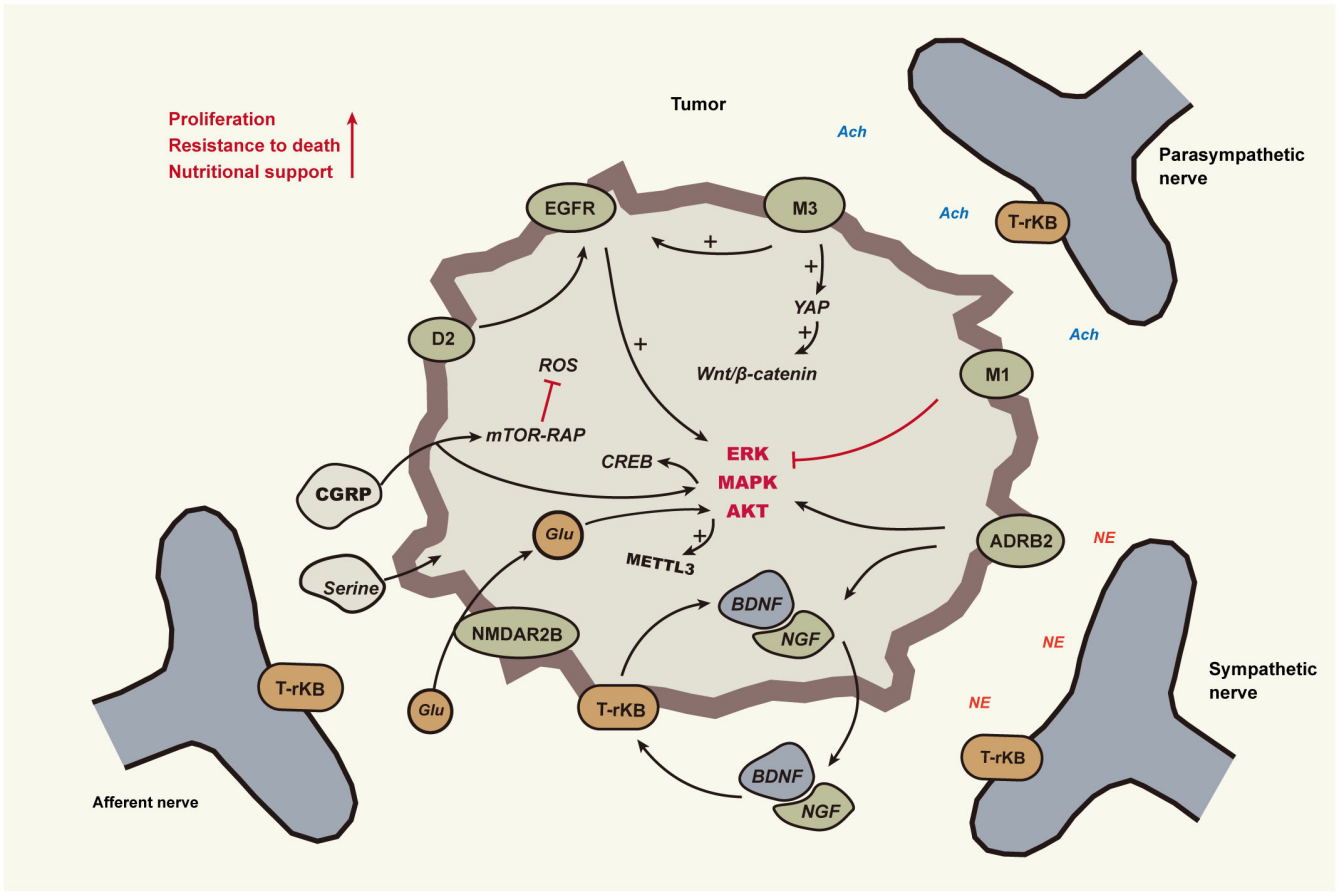
Nerves promote tumour cell growth and survival The neural impact on tumors is mediated through sympathetic, parasympathetic, and sensory nerves, as indicated by solid lines in the diagram. Black lines with arrows signify activation, whereas red lines with dashes indicate inhibition. Cholinergic nerves activate ERK1/2 and AKT signaling molecules via M3 cholinergic receptors mediated by EGFR, and inhibition of pancreatic EGFR/MAPK and PI3K/AKT signaling pathways is achieved through M1. Sympathetic nerves, by releasing catecholamines, activate β2-adrenergic receptors (ADRB2) on tumor cells, subsequently stimulating PKA and ERK pathways. This promotes tumor cell proliferation and transformation and induces the secretion of NGF or BDNF. Neurotrophic factors bind to Trk receptors on peripheral nerve endings, promoting neuronal growth and branching. BDNF, through its interaction with TrkB receptors on tumor cells, activates MAP kinase and Akt pathways, enhancing tumor cell survival and proliferation. Dopamine from nerves synergizes with EGFR to activate the ERK pathway, thereby facilitating cell cycle progression and cell survival. Serine and glutamate from nerves assist in tumor cell glycolysis, protein synthesis, and maintaining mitochondrial activity. Abbreviations : Epidermal Growth Factor Receptor(EGFR), β2-Adrenergic Receptors(ADRB2), Nerve Growth Factor(NGF), Brain-Derived Neurotrophic Factor(BDNF), Muscarinic Acetylcholine Receptor M1(M1), Muscarinic Acetylcholine Receptor M3(M3), Dopamine Receptor D2(D2).

This section primarily delves into the influence of nerves on the growth of periampullary cancer cells and their role in promoting tumor progression. Periampullary cancer is predominantly of the adenocarcinoma type. Nerves play a pivotal role in the normal development and regeneration of glandular epithelium and ductal epithelium tissues. Naturally, as these tissues undergo abnormal proliferation and carcinogenesis, the role of nerves becomes a focal point of attention. Numerous clinical studies have explicitly highlighted the significant role of the nervous system in the proliferative processes of many cancers. Nerves modulate tumor growth progression through various mechanisms, including the release of neurotransmitters, interactions between nerves and tumor cells, and the regulation of neurogenic factors (**Figure 2**).

Cholinergic nerves activate the epidermal growth factor receptor (EGFR) and its associated signaling pathways through cholinergic receptors, including nicotinic and muscarinic types, thus stimulating tumor cell growth. This has been verified in cancers such as lung, liver, and colorectal (12). Muscarinic receptor M3 mediates transactivation of EGFR, activating signaling molecules like ERK1/2 and AKT, which promote tumor cell proliferation and invasion. In gastric cancer, acetylcholine activates the Yes-associated protein (YAP) via the M3 muscarinic receptor (M3R) signaling pathway, thereby regulating the Wnt/β-Catenin signaling pathway and promoting cancer cell proliferation (13). YAP1 has the ability to bind with TEAD and recruit nerves by activating the transcription of the neural growth factor NGF, and research indicates a significant correlation between the occurrence of PNI in prostate cancer and the expression level of YAP1 in tumor tissues (14). In mouse models of gastric cancer, surgical or pharmacological denervation (by cutting or injecting botulinum toxin A) significantly slowed tumor progression and inhibited Wnt signaling and tumor stem cell expansion (15). In prostate cancer, CHRM3 activation enhances growth through CaM/CaMKK-mediated Akt phosphorylation (16, 17). Blocking the muscarinic receptor M3 inhibits the proliferation of cholangiocarcinoma cells (18), but cholinergic nerves play a markedly different role in pancreatic cancer. Renz et al. found that vagotomy promotes pancreatic cancer progression, while the activation of acetylcholinergic signaling inhibits growth, mediated by the M1 cholinergic receptor (CHRM1). This receptor can suppress the EGFR/MAPK and PI3K/AKT signaling pathways in pancreatic cancer cells, thereby reducing the number and activity of cancer stem cells and also inhibiting the formation of liver metastases (19).

Unlike the cholinergic signaling pathway, the adrenergic signaling pathway promotes tumor growth in pancreatic cancer. The sympathetic nervous system stimulates tumor cells by releasing catecholamines which activate the β2-adrenergic receptor (ADRB2) on the tumor cells. This in turn activates the PKA and ERK signaling pathways, promoting tumor cell proliferation, transformation, and the secretion of neurotrophic factors such as NGF or BDNF. These neurotrophic factors bind to the Trk receptors on peripheral nerve endings, enhancing neuronal growth and branching, creating a positive feedback loop. This further stimulates the ADRB2 signaling in tumor cells, accelerating tumor development (20). Sensory nerves play a vital role in pancreatic cancer proliferation and anti-apoptosis. As early as the PanIN stage, they establish a bidirectional signaling circuit with the pancreas, promoting neuritis and tumor formation (21). Additionally, co-cultivation experiments of sensory nerve cells with cancer cells have shown an upregulation of pro-survival genes, such as MALT1 and TRAF, in the tumor cells (22).

As illustrated by the aforementioned examples, within the same neural innervation environment, tumor cells originating from different tissues exhibit varied responses. Moreover, within the same tumor milieu, distinct branches of the nervous system also display different effects. As the natural evolution of a tumor progresses, the tumor cells gradually enhance their capability to proliferate continuously within the adaptive environment. Concurrently, neurons, as a component of the tumor microenvironment, evolve alongside tumor progression, gradually transitioning into forms that are more conducive to tumor cell growth. This phenomenon may epitomize the nature of neural remodeling. In head and neck cancers, sensory nerves are influenced by tumor cells deficient in p53, undergoing a transformation towards sympathetic nerves via extracellular vesicle (EV) transmitted miRNA signaling. These nerves express TH and secrete NA, stimulating tumor growth, invasion, and angiogenesis, thereby augmenting the malignancy of the tumor. Additionally, the removal of sensory nerves can also inhibit tumor growth (23).

Aside from acetylcholine and catecholamines, both neuronal and tumor cells produce cytokines capable of fostering both tumor and neural growth. These factors are instrumental in the interaction between neural and cancer cells. In several malignant tumors, including pancreatic cancer, the brain-derived neurotrophic factor (BDNF) functions by binding to the TrkB receptor, subsequently activating the MAP kinase and Akt signaling pathways. This action bolsters tumor cell survival and proliferation, becoming particularly pronounced when the tumor microenvironment is deficient in growth factors (24–26). Both neuronal and tumor cells release dopamine and serotonin. In cholangiocarcinoma, these compounds facilitate tumor growth. While research on dopamine tends to focus on its secretion and degradation mechanisms within tumor cells, the influence of neural fibers in this process should not be underestimated (27, 28).

## Empowering tumor cells to thrive

Tumors of high malignancy exhibit a notable characteristic: they demonstrate a heightened adaptability to harsh survival conditions compared to other cells. As tumors grow to a certain size, they often face nutritional deficiencies. This leads to spontaneous programmed cell death due to the lack of an appropriate nutritional environment. Moreover, once tumors enter the metastatic phase and depart from their local microenvironment, they must overcome the pressures of anoikis, or detachment-induced apoptosis. Tumors employ various mechanisms to combat these survival stresses, and one such method involves interacting with neural signals.

Dopamine exerts a proliferative effect on glioblastoma cells via the activation of the dopamine receptor D2 (DRD2). In tandem, it exhibits a synergistic interaction with the epidermal growth factor receptor (EGFR), co-activating the ERK signaling cascade, which in turn supports cell cycle progression and promotes cellular viability (29). Intriguingly, upon blockade of the DRD4 receptor, there is a discernible decrement in the activity of key signaling pathways, notably ERK1/2 and mTOR. This results in the stagnation of GNS cells at the G0/G1 phase and a subsequent induction of apoptosis (30). As alluded to earlier, the engagement of BDNF with its cognate TrkB receptor initiates the activation of a series of intracellular signaling pathways. This not only bolsters the resilience and survival capability of tumor cells but also fosters cell cycle advancement and curtails apoptotic events (5, 31). In prostate cancer research, cancer cells with PNI have been found to have a lower apoptosis index, which is closely associated with their enhanced expression of NF-κB and its downstream genes DAD-1 and PIM-2. These molecular alterations within the cells further suggest that, within the context of PNI, the NF-κB signaling pathway might play a crucial role, potentially enhancing cancer cell survival and proliferation rates by inhibiting autophagy (32).

Tumor progression necessitates an abundant supply of nutrients. Moreover, anti-tumor treatments might precipitate nutritional deficiencies, making tumor cells more reliant on support from other cells within the microenvironment. Approximately 40% of PDAC cells lack enzyme expression for the serine biosynthesis pathway (SBP), thus relying on exogenous serine for protein synthesis and mitochondrial activity. In nutritionally deprived conditions, axons of neurons release serine, supporting the metabolism and growth of pancreatic ductal adenocarcinoma (PDAC) cells (33). Additionally, neuronal cells release glutamate, which upon activating the NMDAR2B receptor in PDAC cells, triggers calcium signaling and the CaMKII/ERK-MAPK pathway. This upregulates METTL3 expression and increases the m6A modification of HK2 mRNA, thereby enhancing the glycolytic capability of pancreatic cancer cells. Concurrently, pancreatic cancer cells regulate neuronal growth and amino acid release by secreting NGF and IGF-1, establishing a comprehensive feedback loop (34). Cancer cells undergoing cerebral metastasis can establish connections with glutamatergic neurons to obtain glutamate, stimulating NMDAR signaling and promoting tumor cell colonization (35). However, for other cancer types, this function warrants further exploration. The influence of neuronal cells on other cells within the tumor microenvironment aids tumor survival under nutrient-deficient conditions. Adrenergic nerves modulate endothelial cell metabolism through the ADRB2 signal, promoting angiogenesis and tumor growth (36). Nociceptive nerves secrete calcitonin gene-related peptide (CGRP), interfering with mTOR-Raptor interactions via Rap1-GTPase signaling, inducing autophagy. This aids cancer cells in clearing ROS (reactive oxygen species), maintaining energy equilibrium, and enhancing resistance to nutritional deficiency (37). In the conventional tissue environment of pancreatic cancer, CGRP exhibits a high degree of methylation, affecting the AKT-CREB signaling pathway, further fostering pancreatic cancer initiation and progression (38).

## Nerve-assisted evasion of anti-tumor immunity

**Figure 3:**
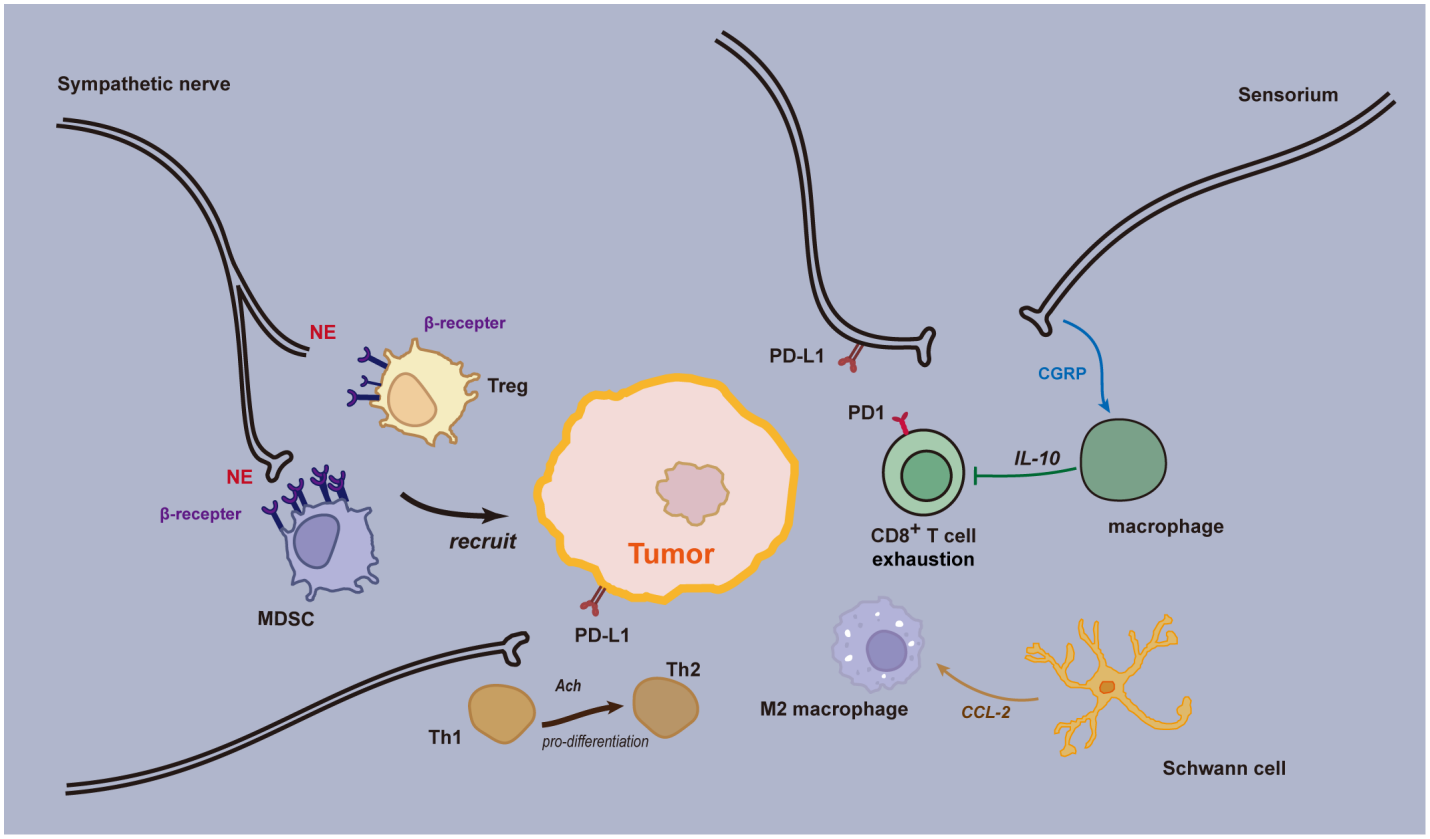
Neural immune regulation in the tumor microenvironment. Sensory neurons secrete CGRP, which acts through the RAMP1 signaling pathway, playing a role in both innate and adaptive inflammatory responses. Upon activation of the RAMP1 receptor, CGRP causes macrophages to release the anti-inflammatory cytokine interleukin (IL)-10, resulting in a reduction of effector CD8+ T lymphocytes. Adrenergic stress from sympathetic nerves leads to the release of norepinephrine and epinephrine, which promote the recruitment of immunosuppressive regulatory T cells (Tregs) and myeloid-derived suppressor cells (MDSCs) to the Tumor Microenvironment (TME). This prevents an effective anti-tumor immune response, facilitating further cancer growth and progression. Some nerve fibers can express immune checkpoint molecules, such as programmed death ligand 1 (PD-L1), which, upon interacting with programmed cell death protein 1 (PD-1), leads to exhaustion of CD8+ T cells in the TME, thereby inhibiting the anti-tumor immune response. Acetylcholine can directly inhibit the production of IFN-γ by CD8+ T cells and promote the shift from Th1 to Th2. Schwann cells (SCs) release chemokine (C-C motif) ligand 2 (CCL2), leading to chemotaxis and specialization of immunosuppressive M2 macrophages. Furthermore, SCs promote the recruitment and immunosuppressive function of myeloid-derived suppressor cells (MDSCs), contributing to cancer progression.

Immune checkpoint blockade has been validated for its therapeutic efficacy across multiple cancer types. Research into the neuro-immune axis has uncovered mechanisms by which the nervous system modulates the residency of immune cells within tissues and the activity of lymphatic immune cells. The nervous system and immune cells often coexist in specific anatomical locations, forming neuro-immune cell units. These units coordinate their respective responses through signals such as neurotransmitters, neuropeptides, and cytokines (39). Within tumors, various neurotransmitters and neuropeptides released by the peripheral nervous system can either directly or indirectly influence the phenotype and behavior of cancer cells, thereby affecting tumor growth and metastasis (**Figure 3**). These factors can also modulate the function of immune cells in the tumor microenvironment, leading to immune suppression and tolerance (40). Neuro-immune modulation in gastrointestinal tumors primarily involves two types: one is through neural stimulation (i.e., neurotransmission), which includes nociceptive signaling, adrenergic signaling, and cholinergic signaling. The other involves the expression of immune checkpoint molecules by tumor-associated nerves and glial cells, such as the PD-1/PD-L1 interaction (41).

A study examining the immune response following radiotherapy across various cancers revealed that chronic adrenergic neuronal stress suppresses both the efficacy of radiotherapy and the anti-tumor immunity. Remarkably, the blockade of this adrenergic stress significantly enhanced the therapeutic control of radiotherapy over tumors (42). This effect is believed to be mediated by augmenting the cytotoxic capabilities and migratory capacity of CD8+ T cells, subsequently leading to an increased expression of effector molecules within the tumor, such as IFNγ, GzmB, TNFα, and T-bet. The blockade of adrenergic neuronal stress also reduces the proportion of M2 macrophages and regulatory T cells within the tumor; both these cell types play roles in immunosuppression and tumor promotion. Moreover, inhibiting adrenergic neuronal stress lowers the expression of co-inhibitory receptors—like CTLA-4, PD-1, LAG-3, and Tim-3—on CD8+ T cells located in non-irradiated regions of the tumor. These receptors limit the effector functions of CD8+ T cells. In a mouse lung cancer model, it was observed that the depletion of tumor-associated macrophages combined with anti-PD-1 immunotherapy restored the migration and infiltration of cytotoxic T cells (43).

Adrenergic and sensory neurons regulate lymph nodes through the β2-adrenergic receptor signaling pathway, controlling the egress of T cells from the lymph nodes, which subsequently leads to a rapid decrease in the number of lymphocytes in the blood and lymphatic fluid. This phenomenon is primarily attributed to the activation of this signaling pathway enhancing lymphocyte retention within the lymph nodes and suppressing their outflow (44). Within peripheral lymph nodes, a subtype of CD8+ memory effector cells, known as T Immune Effector cells (TIE), has been identified. A study on melanoma revealed that in patients treated with immune checkpoint inhibitors, this cell subtype proliferated and was associated with clinical outcomes (45). Post PD-1 blockade treatment, another investigation found clonal expansion of CD8+ T cells within tumors. Interestingly, these T cells were not derived from the original tumor-infiltrating T cells but from new clonal types that hadn’t been previously observed within the same tumor. It is plausible that these novel T cell clones might originate from outside the tumor, such as from the lymph nodes (46, 47). Furthermore, research in mouse models has demonstrated that the combined treatment of sympathetic nerves and CTLA-4 blockers significantly suppressed tumor growth and enhanced the infiltration of CD8+ T cells at the tumor site (48). Based on these findings, we hypothesize that the clonal expansion of peripheral T cells and their infiltration into tumors are critical factors influencing the clinical efficacy of immune checkpoint inhibitors. Additionally, adrenergic neuronal signaling may play a pivotal role in controlling the egress of T cells from the lymph nodes.

Apart from the lymph nodes, lymphocyte aggregates found at the tumor margins, known as Tertiary Lymphoid Structures (TLS), are also influenced by neural fibers. TLS represents ectopic lymphoid organs present in inflamed tissues and cancers. A study discovered that in pancreatic cancer, small nerve fibers are primarily distributed at the tumor margins, spatially co-existing with TLS. Notably, a high density of these small nerve fibers correlates with a more favorable survival prognosis (49). This might be attributed to the small nerve fibers within the tumor microenvironment modulating B cells, thereby influencing the anti-tumor immunity. Upon immune activation, B cells are capable of synthesizing and secreting the neurotransmitter GABA. The GABA secreted by these cells promotes the differentiation of monocytes into anti-inflammatory macrophages that secrete IL-10. These macrophages, through the GABAA receptor signaling, inhibit the TNF signaling pathway, consequently suppressing the cytotoxic function of CD8+ T cells (50). 5-hydroxytryptamine, or serotonin, is another neurotransmitter that can be released by non-neuronal cells. In pancreatic cancer models, serotonin released by platelets can inhibit the effector functions of CD8+ T cells. Furthermore, through serotonergic mechanisms, it stimulates tumor cells to express PD-L1, thus suppressing anti-tumor immunity (51).

The role of acetylcholine in tumor immunity represents an emerging field of research. Recent studies indicate that acetylcholine plays a significant part in modulating the functions of the body’s immune cells. Based on the findings from a clinical trial involving 106 patients, those with advanced solid tumors undergoing pembrolizumab treatment demonstrated improved prognoses when elevated serum choline levels were observed. This suggests that the acetylcholine pathway may have implications in modulating anti-tumor immune responses (52). In the context of thyroid cancer, acetylcholine can induce the expression of PD-L1 in tumor stem cells through the activation of the CD133-Akt pathway, thereby augmenting their resistance against CTLs (Cytotoxic T Lymphocytes) (53).

The levels of acetylcholine correlate with the severity of PNI in pancreatic ductal adenocarcinoma (PDAC). Tissues with pronounced PNI demonstrate significantly elevated concentrations of acetylcholine (54). PNI can modulate the tumor immune microenvironment by releasing acetylcholine through the vagus nerve, subsequently promoting tumor growth. Acetylcholine reduces the recruitment of CD8+ T cells by inhibiting tumor cell secretion of CCL5. This mechanism relies on histone deacetylation mediated by HDAC1. Furthermore, acetylcholine can directly suppress the production of IFN-γ in CD8+ T cells and promote the transition from Th1 to Th2. In animal models, activating or blocking cholinergic signals can regulate the infiltration and differentiation of immune cells within tumors, impacting tumor growth and survival. Vagotomy, or the surgical cutting of the vagus nerve, can enhance CD8+ T cell infiltration, increase the Th1/Th2 ratio, and improve mouse survival (54).

In addition to the autonomic nervous system, sensory nerves also play a role in anti-tumor immunity. Calcitonin Gene-Related Peptide (CGRP) is a neuropeptide, primarily secreted by sensory neurons and serves as a crucial neurotransmitter in the neuro-immune axis (55), playing a negative regulatory role in infection-related immunity (56, 57). The CGRP receptor is composed of RAMP1 and CALCR1 and is present in various malignant tumor cells and tumor-associated immune cells (58). Within the tumor microenvironment, the interaction between cancer cells and sensory neurons often promotes the release of neurotransmitters like CGRP (59). By binding with RAMP1, it leads to functional exhaustion of CD8+ T cells and a reduction in their anti-tumor activity. Genetic knockout or blockage of the CGRP-RAMP1 pathway significantly inhibits tumor growth, and there is a notable increase in the infiltration of CD4+, CD8+, and NK1.1+ lymphocytes in the tumor tissue, enhancing the effectiveness of immune checkpoint inhibitors (58).

In summary, various neural cells and neurotransmitters impact the infiltration of CD8+ T cells in the tumor microenvironment, influencing anti-tumor immunity. These discoveries are significant for understanding why immune checkpoint inhibitors may underperform in “cold” tumors, potentially addressing the immune resistance caused by insufficient CD8+ T cell presence in the tumor microenvironment (60). In peri-visceral tumors, especially in pancreatic cancer, the tumor microenvironment exhibits an immune-suppressed phenotype by promoting immune evasion and limiting anti-tumor immune responses.

### Neural mediation of tumor invasion and metastasis

**Figure 4:**
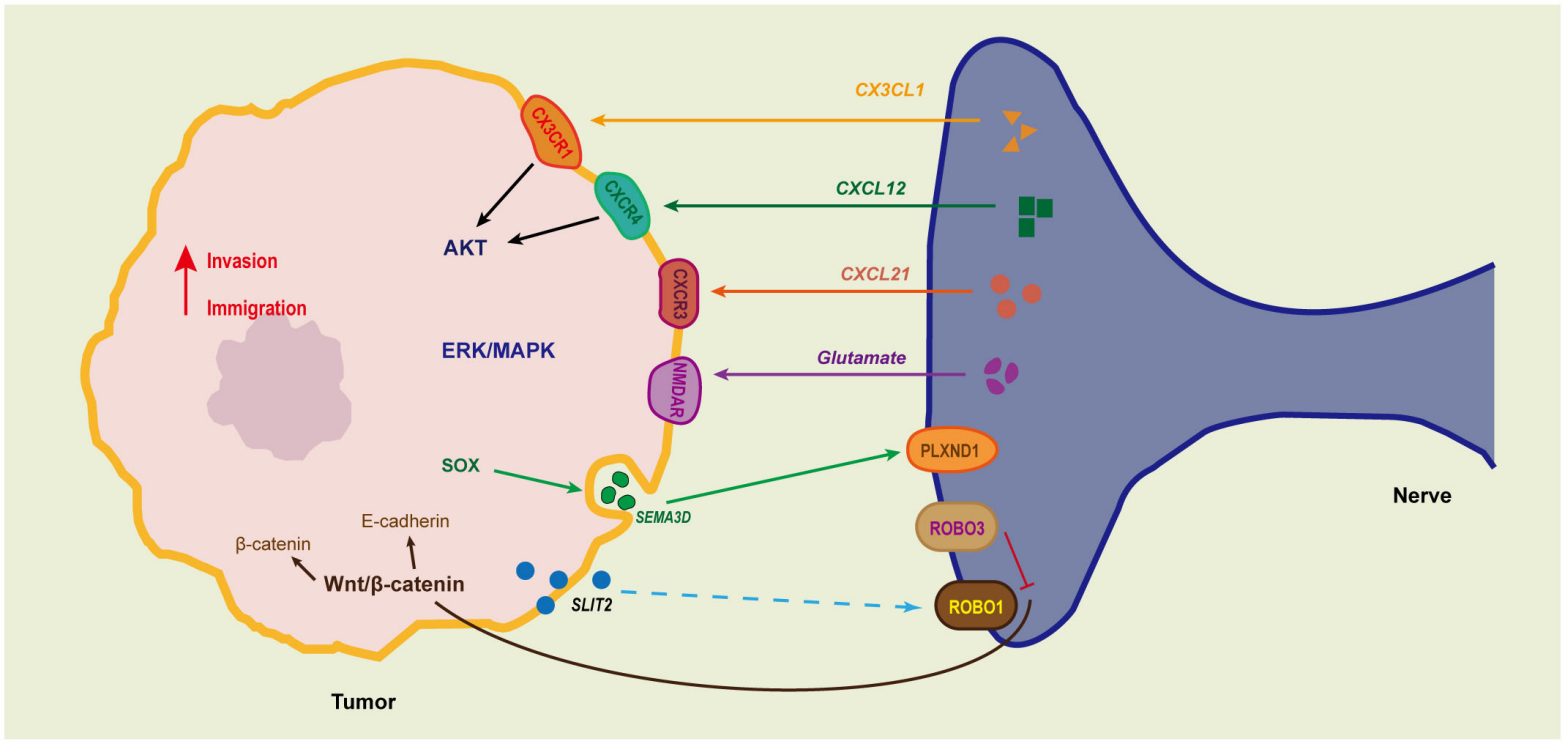
The communication between tumor cells and nerves facilitates tumor perineural invasion. Several mediators are involved in the intricate cancer-neuron communication, including chemokines (such as CX3CL1, CXCL10, CCL21), SLIT2, and the axon guidance molecule SEMA3D. CX3CL1, highly expressed in various neurons, plays a role in the adhesion between nerve cells and stromal cells. This adhesion is enhanced when tumor cells express specific adhesion molecules, promoting tumor migration towards nerves. Peripheral nerves secrete CXCL12, which, through its receptor CXCR4, can upregulate invasion-related genes like MMP-2 and uPA, as well as the AKT signaling pathway, thereby facilitating the migration and invasion of pancreatic cancer cells. Additionally, the CXCL10-CXCR3 and CCL21-CCR7 chemokine axes regulate pancreatic cancer cell migration via the AKT and ERK signaling pathways. The secretion of the axon guidance molecule SEMA3D, through the NGF-TRKA and ANAX2-SEMA3D-PLXND1 signaling axes, promotes bidirectional chemotaxis between nerves and tumors, narrowing the distance between tumor cells and nerves.

For malignant tumors, one of the greatest risks is their ability to detach from the primary site, enter the circulatory system, and form metastatic lesions in other areas. This also stands as one of the primary challenges in tumor therapy. Nerves within tumors promote the growth and migration of tumor cells in various ways, including through interaction with tumor cells via neurotransmitters and neurotrophic factors, as well as serving as physical pathways for cancer cell migration through their fibrous structure (61). The nerves inside the tumor might originate from the peripheral nervous system, central nervous progenitor cells, or tumor stem cells. These nerve fibers could be guided into the tumor tissue by chemotactic factors secreted by the tumor, or result from tumor cells infiltrating and surrounding existing nerves. In some cases, both phenomena might occur simultaneously, resulting in an aberrant nerve fiber presence within the tumor (**Figure 4**). Current research on the interaction between nerves and tumors in Periampullary cancer mainly focuses on the concept of PNI. Pathologically, PNI indicates that tumor cells have infiltrated the neural sheath. However, once tumor cells migrate closer to nerve cells, they can more easily harness the support of nerves for tumor cell growth, immune evasion, invasion, and migration, even acting as mediators for vascular and lymphatic metastases. Research has found that the surface of cholangiocarcinoma contains more nerve fibers than normal bile duct walls. These fine fibers are connected to deeper, thicker nerve fibers and infiltrate along these fine fibers, gradually enveloping and expanding around the coarse fibers (62). The invasion of extrahepatic cholangiocarcinoma cells into surrounding tissues is notably correlated with PNI and is not dependent on blood supply (10). In pancreatic ductal adenocarcinoma, the occurrence of PNI is significantly associated with post-surgical recurrence due to microvascular invasion (63). Moreover, studies have indicated that PNI is an early event in tumorigenesis, underscoring its significance in the early metastasis mechanisms within tumors (64).

In prostate cancer, cholinergic signaling promotes the Hh signaling pathway through the stimulation of CHRM1, thereby enhancing the invasiveness and metastatic capabilities of cells. Moreover, CHRM1-specific antagonists can counteract this effect (65). Cigarette smoke extract (CSE) and nicotine activate the JAK2/STAT3 and Ras/Raf/MEK/ERK1/2 signaling pathways through the α7 subunit of the nicotinic acetylcholine receptor (α7nAChR). This activation significantly increases the transcription and expression of MUC4 mucin in pancreatic cancer cells, thereby promoting tumor metastasis (66). However, intriguingly, in pancreatic cancer, the activation of CHRM1 can inhibit the growth and migration of tumor cells (19). This suggests that even for the same neurotransmitter, its effects might vary depending on the receptors it interacts with. While the parasympathetic nervous system generally suppresses the growth and development of pancreatic cancer, its effects at the cellular level might differ.

Sympathetic neurotransmitter norepinephrine (NE) activates the ADRB2/PKA/STAT3 signaling pathway, leading to an increase in pancreatic cancer cell expression of NGF, MMP2, and MMP9, thereby enhancing their migration, invasion, and nerve infiltration capabilities (67). Under chronic stress conditions, the activation of sympathetic nerve signals acts on stromal cells, further amplifying the VEGFC-VEGFR3 signal transmission (68). In addition, NE can also act on tumor cells, modulating the STAT3 signaling pathway, which impacts tumor angiogenesis (67). This process leads to alterations in the vasculature and lymphatic structures surrounding the tumor. Therefore, it can be inferred that the nervous system not only serves as a physical pathway for tumor metastasis but also plays a role in regulating the formation of tumor blood and lymphatic vessels, providing more avenues for tumor dissemination. During the normal tissue development process, angiogenesis is closely linked with neural regulation. BDNF, while primarily known as a neurotrophic factor, is also considered a potential angiogenic factor. BDNF interacts with its receptor, TrkB, regulating endothelial cell proliferation, migration, invasion, and survival. It also modulates the expression of TrkB and VEGF in tumor cells, promoting angiogenesis and related tumorigenic processes (69).

In the peri-pancreatic tissue, various cells assist in the interaction between nerves and tumors and promote tumor growth and metastasis. Apart from various neural cells, Schwann cells, the most vital glial cells in the peripheral nervous system, play a significant role. When tumor cells invade peripheral nerves, Schwann cells undergo reprogramming under the influence of tumor cells, transforming into tumor-associated Schwann cells (TSCs). In melanoma, tumor cells induce Schwann cells to undergo degenerative changes and repair reactions, converting them into repair-type Schwann cells (rSCs). These rSCs exhibit enhanced extracellular matrix remodeling, cell migration, macrophage recruitment, and polarization, all of which create favorable conditions for the growth and spread of melanoma (70). Under the regulation of tumor cells, Schwann cells activate autophagy, which is crucial for tumor cell invasion into nerve fibers and further metastasis. Pancreatic cancer cells secrete nerve growth factor (NGF), impacting Schwann cells. Through the p76NTR/AMPK/mTOR signaling pathway, autophagy is activated in Schwann cells, enhancing their ability to phagocytose and degrade cellular debris and increasing their chemotaxis towards tumor cells (71, 72). This creates a bridge for tumor cells to invade nerve fibers. Moreover, Schwann cells promote the migration and invasion of PDAC cells by secreting the chemokine CCL7 and activating the CCR2/STAT3 signaling pathway. This also upregulates the expression of tissue inhibitor of metalloproteinase 1 (TIMP 1) in PDAC cells. Further, through the activation of the CD63/PI3K/AKT signaling pathway, TIMP1 activates SCs, forming a cytokine feedback loop between PDAC cells and SCs (73). As a cancer-associated cytokine, TIMP1 influences tumor proliferation and metastasis, offering a potential therapeutic target for pancreatic cancer. Schwann cells also secrete the chemokine CCL2, attracting monocyte (IM) migration. Subsequently, these monocytes differentiate into macrophages and produce cathepsin B (CTSB). CTSB can degrade type IV collagen in the perineurium, compromising its integrity and thus enhancing the likelihood of tumor cell invasion (74).

Similar to Schwann cells, sensory nerves can also express chemokines that promote tumor cell invasion and metastasis, and under the influence of NGF, they extend towards tumors. The transmembrane chemokine CX3CL1, which is highly expressed in various neurons, plays a role in the adhesion process between neural cells and matrix cells. When tumor cells express specific adhesion molecules, this adhesive effect is enhanced, promoting tumor migration towards nerves. During the adhesion process between PDAC cells and nerve cells, the CX3CR1 receptor activates the FAK signaling pathway through both G-protein-dependent and β1 integrin-dependent mechanisms (75). This strengthens the stable binding between tumor cells and nerves, further encouraging tumor migration towards the neural direction. Peripheral nerves release CXCL12, which, through its receptor CXCR4, upregulates invasion-associated genes like MMP-2 and uPA and activates the AKT signaling pathway (76, 77). This promotes the migration and invasion of pancreatic cancer cells. Furthermore, the chemokine axes CXCL10-CXCR3 and CCL21-CCR7 can also regulate the migration of pancreatic cancer cells through the AKT and ERK signaling pathways (78).

Neural and tumor cells exhibit mutual chemotactic interactions, with each entity promoting the growth and migration of the other. This interplay often results in tumor cells migrating along nerve fibers, a phenomenon termed PNI. This mutual chemotactic effect can be facilitated through various chemokines, signaling molecules that direct the movement of diverse cell types (**Figure 4**). The chemokine CXCL12 is released from sensory neurons and binds to receptors CXCR4 and CXCR7 on tumor cells. Activation of the CXCL12/CXCR4 axis significantly not only increases perineural invasion (PNI) in PDAC cells but also promotes the growth of nerve axons (76). Within pancreatic cancer cells, there is secretion of the Nerve Growth Factor (NGF) and axon guidance molecule SEMA3D. These factors, via the NGF-TRKA and ANAX2-SEMA3D-PLXND1 signaling axes, respectively, enhance the bidirectional chemotaxis between nerves and tumor cells, bringing them in closer proximity (79, 80). Furthermore, the role of NGF within the pancreatic cancer microenvironment is multifaceted. Both pancreatic cancer cells and pancreatic stellate cells are capable of secreting NGF. Activation of the NGF-TRKA pathway, through the PI3K/AKT/GSK signaling cascade, augments the proliferative and invasive capacities of pancreatic cancer cells (79, 81). Research indicates that in both pancreatic and breast cancer models, blocking the Nerve Growth Factor (NGF) effectively counters tumor invasion and metastasis (82, 83).

The Slit/Robo signalling pathway is also a signalling pathway that originally regulates axon guidance in the nervous system, consisting of Slit ligands and Robo receptors, and plays an important role in the genesis, development and metastasis of a wide range of tumours. SLIT2 can hinder both the reciprocal chemotaxis between pancreatic cancer cells and nerve cells as well as the directed migration of pancreatic cancer cells along nerve axons, either through self-secretion or paracrine mechanism mediated by ROBO1 (84). Research has shown that the expression of Slit2 is downregulated in pancreatic cancer tissue, whereas the expression of Robo1 is upregulated. Knocking out Robo1 can prevent the invasion of pancreatic cancer into the surrounding nerves (85). Additionally, the SLIT2-ROBO1 axis is critical in the liver metastasis of pancreatic ductal adenocarcinoma (PDAC). Liver cells possess the ability to secrete SLIT2 and interact with ROBO1-positive PDAC cells, which can further stimulate the growth and implantation of PDAC cells in the liver (86). Antibodies that target the SLIT2-ROBO1 axis can prevent SLIT2 from binding to ROBO1, which inhibits activation of the p38α MAPK pathway and triggers ROBO1-driven apoptosis (85, 86). This approach holds promise as a strategy for combating invasion and metastasis in pancreatic cancer.

## Neuroinflammation and neuropathic pain in tumors

Research has shown that in pancreatic ductal adenocarcinoma (PDAC) tissues, there is a notable reduction in neural density and the number of nerves compared to normal pancreatic tissues. Concurrently, nerve fibers in PDAC tissues manifest hypertrophy and inflammation. An increase in the quantity and diameter of pancreatic nerve fibers is initially observed in patients with chronic pancreatitis (87). As chronic pancreatitis evolves into malignant pancreatic tumors, there is a progressive remodeling of the neural architecture, which plays a significant role in the development and progression of malignant tumors.As the tumor progresses, there is a gradual reduction of nerve fibers within PDAC tissues, especially in the tumor core, which becomes filled with fibrotic tissue. Both a reduced neural density and a high proportion of neural invasion are independent factors indicative of a poor prognosis for PDAC patients. In PDAC tissues with low neural density, a higher proportion of cancer cells invade nerve fibers, while in tissues with higher neural density, there is a lower proportion of such invasion (88). This could be attributed to the assault on nerve fibers by cancer cells in PDAC tissues, leading to neural damage and degeneration, resulting in a decrease in neural density and number. This phenomenon constitutes an essential aspect of pancreatic cancer neural plasticity. Pancreatic neural plasticity refers to the morphological and functional alterations of the pancreatic nerves in conditions like chronic pancreatitis and pancreatic cancer, encompassing nerve proliferation, hypertrophy, inflammation, and invasion. These changes might be associated with neuropathic pain. Moreover, neural plasticity might also impact the activity of the central nervous system, leading to hypersensitivity and structural alterations in the spinal cord and cerebral cortex, intensifying pain perception (89).

A clinical study involving 546 patients revealed that in pancreatic cancer, the most evident morphological alterations in nerves are tumor cell invasion and neuropathic inflammation, both closely associated with abdominal pain symptoms (90). Neural inflammation might arise from the assault and damage inflicted by inflammatory cells or tumor cells, stimulating and activating pancreatic afferent fibers and pain-associated structures in the central nervous system. In patients with chronic pancreatitis, neural structures undergo destruction and remodeling. The neural sheath encasing nerve bundles sustains damage, manifesting as degeneration and disintegration. Consequently, it fails to effectively form a protective barrier, cannot maintain normal ion concentrations, osmotic pressure, or fluid movement, nor can it shield the microenvironment within the nerves. Furthermore, the nerves are influenced by various substances within the surrounding connective tissue space, including plasma components and bioactive substances released by inflammatory cells (87). These factors could lead to anomalies in the generation and transmission of neural impulses, exacerbating pain symptoms in patients. With respect to the types of nerves, there’s a significant reduction of sympathetic nerve fibers in tissues from both chronic pancreatitis and pancreatic cancer. Especially in patients exhibiting pronounced pain symptoms, the proportion of sympathetic and parasympathetic nerve fibers in the total nerve area is notably lower than in patients without pain symptoms (91). Although there’s an overall decrease in autonomic nerve numbers in tumor tissues, a marked difference exists between sympathetic and parasympathetic nerves. This distinction might be tied to their individual interactions with tumor cells; for instance, in pancreatic cancer, sympathetic and parasympathetic nerves might have diametrically opposing roles (19, 20). As a result, tumor cells tend to invade in directions that are more favorable for their growth, moving physically closer to these nerves.

Tumor cells promote Schwann cell autophagy via paracrine signaling and attract monocytes, subsequently creating an inflammatory environment around the nerves that fosters tumor growth (71, 72). This process simultaneously results in damage and functional impairment of neural cells and their myelin sheaths. On one hand, neural inflammation may provide a favorable microenvironment for tumor cells, enhancing their migration and survival. On the other hand, tumor invasion into nerves might lead to neural damage and heightened sensitivity, inducing cancer-related pain and other neurological symptoms (92). Consequently, once perineural invasion occurs, tumor cells manage to overcome various stresses in their proliferation and metastasis processes through interaction with nerves. Meanwhile, nerve fibers within the tumor tissues undergo degradation, diminishing in number. Nerves that engage in more intimate interactions with the tumor suffer greater damage. Hence, there are fewer autonomic nerves found within the tumor microenvironment compared to a relatively higher number of sensory nerves. However, mouse experiments have revealed that the density of tumor sensory nerve fibers and sympathetic nerve fibers increases with tumor growth, primarily localizing around the tumor periphery and adjacent to blood vessels (93). Perhaps a dynamic equilibrium exists between the invasive destruction of nerve fibers by tumor cells and the promotion of nerve growth and extension due to the combined effects of various cells within the tumor microenvironment. This might also explain why early-stage pancreatic cancer often presents without significant pain symptoms, whereas advanced stages are characterized by intense pain.

Pain resulting from neural inflammation can be considered a common feature of both chronic pancreatitis and pancreatic tumors. However, the pain induced by malignant tumors may be more severe, possibly for other reasons. Pancreatitis itself is a significant risk factor for pancreatic cancer, especially in cases of chronic pancreatitis and hereditary pancreatitis. They share certain pathogenic mechanisms, such as oxidative stress, inflammatory response, cell apoptosis, and genetic mutations (94). Furthermore, some studies suggest that the carcinogenesis of human pancreatic tissue is jointly caused by genetic alterations and non-genetic factors represented by chronic pancreatitis (95). Therefore, in the context of pancreatic cancer, interactions between tumor cells and sensory nerves, rooted in an inflammatory tissue environment, might lead to exacerbated pain symptoms. Sensory neurons trigger neurogenic inflammation by releasing neuropeptides like substance P, which play a pro-inflammatory role in the onset and persistence of pancreatitis. In the early stages of pancreatic ductal adenocarcinoma (PDAC), specifically during the PanIN phase, pancreatic cells invade the spinal cord and peripheral ganglia along sensory nerves, eliciting inflammatory responses and neuronal damage in the spinal cord (21). These changes might contribute to the onset of chronic pain and malaise.

The role of nerve growth factor (NGF) in the pain mechanism of tumors is increasingly drawing the attention of researchers, especially in pancreatic-related diseases. NGF not only affects neural development and survival but also participates in the regulation of inflammation and hypersensitivity, such as promoting histamine release from mast cells and enhancing pain sensitivity. In studies on chronic pancreatitis, data reveal a significant upward trend in the expression of nerve growth factor and its high-affinity receptor, TrkA. This expression is primarily concentrated in key areas such as degenerated pancreatic acinar cells, ductalized pancreatic acinar cells, ductal epithelial cells, nerve fibers, and vessel walls. Notably, levels of NGF mRNA correlate positively with the degree of pancreatic fibrosis, damage to pancreatic acinar cells, and the extent of ductalization. Similarly, levels of TrkA mRNA correlate positively with pain intensity (96). These findings suggest that NGF may play a pivotal regulatory role in inflammatory responses and pain transmission. As NGF expression intensifies, its binding to receptors could activate specific signaling pathways, thereby amplifying pain transmission and perception.

Pain associated with pancreatic cancer is intricately linked to numerous biological mechanisms. This involves alterations in tumor vasculature, active infiltration of macrophages, and aberrant innervation of nerves. Notably, both macrophages and tumor cells possess the capability to express NGF, which in turn can induce the growth and extension of nerve fibers (93). Such alterations might trigger abnormal neural activity, leading to the onset of pain. In this process, NGF may modulate intercellular interactions by binding to its specific receptors, further intensifying or alleviating pain perception (64). Of interest, neural plasticity changes have already emerged in the early stages of pancreatic ductal adenocarcinoma. These early neural changes are potentially directly associated with nerve growth factor (NGF), wherein NGF may regulate the growth and differentiation of neural cells, making them more sensitive to pain signals (97). Moreover, the Sonic hedgehog signaling pathway also plays a central role in the formation mechanism of tumor pain. Studies have found that activation of this signaling pathway can elevate the expression levels of NGF and its receptors in DRG cells, which then promotes the expression and secretion of SP and CGRP, exacerbating the pain of pancreatic cancer (98).

TRPV1, known as the Transient Receptor Potential Vanilloid 1, is a predominant acid-sensitive pain receptor primarily expressed in sensory neurons. It can be activated by capsaicin, heat, and acidic conditions. This receptor has garnered significant attention in both the fields of biology and medicine due to its multifaceted roles. In recent years, researchers have delved deep into the role of TRPV1 in the context of tumorigenesis, particularly its involvement in tumor initiation, progression, and treatment modalities. In pancreatic cancer cells, TRPV1 exhibits a regulatory effect on the Epidermal Growth Factor Receptor (EGFR). Specifically, TRPV1 promotes the ubiquitination of EGFR, subsequently modulating the EGFR/MAPK signaling pathway. When activated, TRPV1 can influence a multitude of biological processes, including pH regulation, temperature sensing, and interaction with calcium ions (Ca2+). These mechanisms are potentially correlated with the proliferation, invasion, and migration of tumor cells. Further studies underscore the interplay between TRPV1 and the ubiquitination of EGFR, along with subsequent signaling pathways. This interaction may be a pivotal determinant in the aggressive growth and deterioration of pancreatic cancer cells (99). Additionally, in hepatocellular carcinoma, TRPV1 has been demonstrated to modulate cellular plasticity. Research data suggests that TRPV1 orchestrates cell plasticity through mediators like Ovol 2 and Zeb 1. This regulation may be associated with the growth and differentiation of neurons, subsequently affecting the cellular response to pain signals (100). Lung cancer-related studies have unveiled the profound effects of TRPV1 on sensory neuronal excitability and bone pain. In mouse models deficient in TRPV1, both the neuronal excitability and bone pain associated with lung cancer were markedly reduced (101). Contemporary research indicates that by blocking the TRPV1 channel through nanoparticle mediation, the sensitivity of cancers to hyperthermia immunotherapy can be augmented. This finding has been validated across various primary, metastatic, and recurrent tumor models (102). From pancreatic to hepatocellular and then to lung cancer, the implications of TRPV1 intertwine deeply with tumor dynamics, therapeutic strategies, and pain management. With continued exploration into TRPV1, we might witness the emergence of more tumor treatment modalities centered around this pivotal receptor in the future.

## The interplay between neural signaling and cancer stem cells

Cancer stem cells (CSCs) are key drivers of tumor progression and metastasis, endowed with the capacities for self-renewal and differentiation into various tumor cell types. Recent research has increasingly uncovered the critical role of neural components, particularly neurotransmitters and neurotrophic factors, in regulating the self-renewal, differentiation, and the sculpting of the tumor microenvironment of CSCs (103, 104). The nervous system not only provides nutritional support to tumors but also directly promotes the self-renewal and proliferation of CSCs through the secretion of neurotransmitters such as serotonin and acetylcholine.

In a variety of malignant tumors, the interaction between neuronal signaling and cancer stem cells not only stimulates tumor growth but also represents a potential therapeutic target against CSCs (105–107).Numerous studies have demonstrated that neurotrophic factors (such as NGF and BDNF) and their receptors (such as TrkA and p75NTR), neurotransmitters (such as dopamine and acetylcholine), as well as other neural system components, exert direct or indirect regulatory effects on CSCs. These signals facilitate CSCs’ self-renewal, enhance their drug resistance, and influence their differentiation state by activating specific signaling pathways, including PI3K-Akt, Wnt/β-catenin, and YAP/TAZ (108). For instance, in colorectal cancer, serotonin (5-HT) signaling activates the Wnt/β-catenin pathway through the HTR1B/1D/1F receptors, promoting CSC self-renewal (109). In malignant peripheral nerve sheath tumors (MPNSTs), endogenous catecholamines maintain CSCs’ cancer stemness by activating YAP/TAZ (107).

Some studies have revealed that CSCs can differentiate into neuron-like cells under certain conditions, participating in tumor neurogenesis and the formation of nerve fibers. For instance, colon cancer stem cells have been shown to generate neurons that contribute to tumor neurogenesis, and CSCs isolated from gastric cancer patients can produce neurons, including sympathetic and parasympathetic neurons, participating in the neural system of cancer tissues (110, 111). This phenomenon not only enhances our understanding of the complexity of the tumor microenvironment but also opens new avenues for cancer therapy.

The interaction between the nervous system and CSCs is not unidirectional. On one hand, CSCs can influence the growth and distribution of surrounding nerve fibers and even have the capacity to guide nerve fibers into penetrating the tumor microenvironment, promoting tumor neurogenesis. This tumor neurogenesis not only supports tumor growth but also provides the tumor with additional pathways to resist treatment, increasing the complexity of therapy.In terms of treatment, targeting the interactions between nerves and CSCs, such as using serotonin signaling blockers or acetylcholine receptor antagonists, can effectively slow tumor growth and enhance therapeutic outcomes. Moreover, research targeting NGF and its receptors has revealed new ways to regulate the interactions between neurons and CSCs within the tumor microenvironment, offering possibilities for developing new therapeutic strategies (112).

Through these studies, we have not only gained a deeper understanding of the complex interactions between the nervous system and CSCs but also opened new avenues for future cancer treatment. Future research needs to further explore the specific mechanisms of these signals across different tumor types and validate the effectiveness of therapeutic strategies targeting these signaling pathways in clinical settings (53, 113).

## Therapeutic strategies

The tumor microenvironment (TME) holds immense significance in the field of cancer therapy. This importance stems from the fact that the TME not only provides the essential conditions required for the growth, spread, and invasion of cancer cells but also directly impacts the efficacy of treatments and the prognosis for patients. For instance, the poor response of pancreatic ductal adenocarcinoma (PDAC) to current treatments can be attributed to its characteristic TME, which supports cancer cells in resisting immunotherapy and chemotherapy (114).

Nerves constitute an integral part of the tumor microenvironment, where their interaction with tumor cells, fibroblasts, immune cells, and other components is a crucial step in tumor progression. Consequently, therapies targeting nerves may influence outcomes directly or indirectly. There is now a consensus that targeting the bidirectional communication between nerves and cancer can slow tumor growth, suppress various capabilities of tumor cells, and even lead to cure, resulting in improved prognoses (114). Drugs that act on nerves, capable of disrupting neurotransmitter transmission and other neural signals, have been widely applied in tumor treatment experiments and have demonstrated promising efficacy.

Utilizing approved drugs that can interfere with neurotransmitters and various neural signals for the treatment of specific cancers appears to be a viable and rapid pathway for clinical translation.For instance, the sympathetic nervous system plays a central role in tumor growth and progression; thus, numerous studies have focused on exploring the impact of inhibiting sympathetic excitability on tumor treatment. Research indicates that cancer patients who regularly take β-blockers for other conditions typically have better prognoses than those who do not use such medications (115, 116).

Preliminary clinical trial results indicate that beta-blockers can safely modulate sympathetic neural signaling in breast cancer patients, and when combined with neoadjuvant chemotherapy, they demonstrate good tolerability (117). Furthermore, studies highlight that beta-blockers are effective in reducing biomarkers associated with the invasiveness of breast cancer cells, while also enhancing biomarkers related to anti-cancer immunity (117, 118).

Additionally, a significant number of drugs targeting neuro-cancer signaling pathways are currently under development, including NGF (Nerve Growth Factor) inhibitors, Glutamate release inhibitors, and Voltage-Gated Sodium Channel (VGSC) inhibitors (115). Blocking tumor cells’ recruitment of nerves is considered a potential therapeutic target. Larotrectinib, a drug specifically targeting tumors harboring neurotrophic receptor tyrosine kinase (NTRK) gene fusions, has been approved. It effectively inhibits the activity of TRK-NGF, thereby significantly reducing pancreatic tumor growth (33, 119). Moreover, in the field of tumor therapy, monoclonal antibodies against NGF not only effectively suppress tumor growth but also provide analgesic effects (120–122). Given the close association between neurogenesis and angiogenesis, antibodies targeting NGF play a role in combating angiogenesis (82). Additionally, inhibitors of the NF-κB pathway can suppress NGF-mediated perineural invasion (PNI) and nerve growth. Specifically, *in vivo*, inhibiting the NF-κB pathway leads to a reduction in neurotrophic factor expression, nerve density, and PNI, further elucidating the potential mechanisms and importance of these therapeutic approaches in blocking tumor progression (123).ONC201, a small molecule inhibitor of dopamine receptors 2/3 (DRD2/3), not only induces apoptosis and cell cycle arrest in various cancer cells by activating the TRAIL pathway and inhibiting AKT and ERK signaling but also has demonstrated favorable results in pancreatic cancer treatment when used in combination with other drugs (124–126).

Therapeutic approaches targeting the interactions between nerves and cancer may have the potential to act independently. More likely, they could serve as sensitizers to radiotherapy or chemotherapy, or synergize to enhance the efficacy of anti-tumor immunotherapies (127). Consequently, extensive clinical research is needed to explore the optimal combinations of these drugs to achieve the most meaningful clinical outcomes.

## Discussion

PNI is intimately linked to the pre-existing neural fiber network. Pancreatic perineural cancer, due to its unique anatomical features, is especially prone to dissemination via the perineural network. This suggests that therapies targeting neural pathways might be particularly effective for such cancers. However, while PNI is prevalent in pancreatic cancer, it is not manifest in all patients, posing a significant question: Is treatment targeting neural pathways still effective in patients without overt signs of neural invasion? The key lies in the accurate preoperative prediction of potential PNI occurrence. Advances have been made, such as the development of radiomic-based predictive models that forecast the PNI status in cholangiocarcinoma non-invasively, enabling more tailored surgical planning and precise targeted and immunotherapy regimens (128, 129). Yet, when considering therapies targeting neural pathways, potential side effects cannot be overlooked, given the autonomic nervous system’s critical role in digestive functions. Thus, the benefits of such treatments must be carefully weighed against their possible impact on normal physiological functions (130).

Currently, integrating therapies addressing the interplay between nerves and cancer with traditional treatment modalities like surgery and chemotherapy is regarded as offering a more potent synergistic effect. Although these multimodal combined treatment approaches are promising, they require further research and development. A deeper understanding of the role of nerves within the tumor microenvironment and their interaction mechanisms with cancer cells is crucial for developing novel therapeutic strategies. Such knowledge could lead to treatments targeting specific neural signaling pathways to enhance immune responses and promote tumor cell apoptosis, but this necessitates more extensive research support.

In summary, neurons are not merely components of the tumor microenvironment affected by cancer but play a pivotal role in the balance of this milieu. In the tumor microenvironment, neurons serve as a matrix exploited by the tumor, as vital as neoangiogenesis. Tumor cells dominate the interactions between nerves and cancer. The varying incidences of PNI among cancers of different tissue origins highlight the heterogeneity of tumor tropism for nerves (119). This tropism may persist and present as PNI characteristics at metastatic sites, suggesting that therapies targeting neural pathways could not only alleviate pain but also open new avenues for treating advanced-stage cancer patients. As focus intensifies on the intersection of oncology with neuroscience, particularly for tumors with a proclivity for neural invasion like pancreatic and cholangiocarcinoma, future research will undoubtedly concentrate on revealing their interaction mechanisms with nerves.

In prostate cancer, the YAP1/TEAD complex mediates the occurrence of PNI by activating the transcription of the nerve growth factor NGF (14). In pancreatic cancer, the overexpression of YAP1 may promote invasiveness, epithelial-mesenchymal transition (EMT), and resistance to gemcitabine. We hypothesized that YAP1 might also regulate the tropism of tumor cells for nerves in pancreatic cancer, but the experimental outcomes did not meet our expectations. In a sample of 48 pancreatic cancers, YAP1 expression levels did not significantly differ, nor were they notably correlated with the presence of PNI. This might be due to the high incidence of PNI in pancreatic cancer or YAP1 not playing a major role in the neural tropism of pancreatic cancer cells. Notably, the high incidence of PNI in pancreatic cancer signifies a prominent interaction between the tumor and nerves, bearing significant implications for treatment strategies. However, the scarcity of tumor samples without PNI presents challenges for research. Compared to pancreatic cancer, PNI is less frequent in perineural cancer and cancers of other origins, with relatively fewer studies on their mechanisms of neural tropism. Investigating tumors with a moderate rate of PNI might be more revealing in terms of differentiating between the neural affinities of various cancer cells, potentially uncovering critical genes and signaling pathways. Such research would provide deeper insights into these diseases, guiding the development of new treatment methods that not only improve survival rates but also enhance the quality of life for patients.

In conclusion, the intricate interplay between nerves and tumors demands a more comprehensive and precise approach in developing treatment protocols. While therapies targeting neural pathways have shown potential effectiveness in some cases, we must continue to explore which patients are most likely to benefit and develop strategies that minimize interference with normal physiological functions. Furthermore, personalized treatment plans, based on each patient’s unique tumor characteristics and neural system status, may be key to future cancer therapies. Ultimately, by increasing our understanding of the role of neuroscience in oncology, we can look forward to developing more effective, targeted, and less side-effect-prone treatments, offering new hope to patients.

## Author contributions

YW: Writing – original draft. ZL: Writing – original draft. YT: Writing – review & editing. HZ: Writing – review & editing. XF: Writing – review & editing.
